# Chronotype in individuals with exercise addiction – preliminary analysis

**DOI:** 10.1192/j.eurpsy.2025.953

**Published:** 2025-08-26

**Authors:** M. J. Kania, A. J. Krupa, A. Chrobak, M. Siwek

**Affiliations:** 1 Clinical Department of Adult Psychiatry, University Hospital in Krakow; 2 Department of Affective Disorders; 3 Department of Adult Psychiatry, Jagiellonian University Medical College, Krakow, Poland

## Abstract

**Introduction:**

Exercise addiction (EA) is conceptualized as the uncontrolled practice of physical activity, with repetition of that behavior despite the negative effects on the individual [Khazaal et al. Rev Med Suisse. 2024; 20(882):1354-1359]. According to current systems, EA is included in the group of behavioral addictions [Martyniak et al. Psychiatr Pol 2021;55(6):1357-1372]. The prevalence of EA is understudies, with data from athlete samples ranging from 5-17% [Weinstein et al. Dialogues Clin Neurosci. 2023; 25(1): 1–13]. No prior studies investigatedthe links between chronotype and EA.

**Objectives:**

This work aimed to assess the relationship between EA and chronotype.

**Methods:**

This is a preliminary analysis of data from a larger online survey that assessed the links between behavioral addictions and psychopathology in an online community sample. The inclusion criteria were 1) age ≥18-35 and 2) consent to participate. A local bioethical committee approved the study. Responders completed self-report questionnaires to assess EA: EAI (Exercise Addiction Inventory-Short Form), and chronotype Composite Scale of Morningness (CSM). The participants were divided into subgroups based on their EAI scores: 1) with negative EA screen (EA [-]) [scores ≤12], 2) with positive EA screen (EA [+]) [scores >12]. EA[-] and EA [+] were compared in terms of general characteristics and chronotype (continuous measure). The quantitative data were evaluated with a T-test. The qualitative data were analyzed using the Χ2 test.

**Results:**

183 subjects were EA [-] and 99 were EA [+]. There were no significant differences between the groups regarding the mean age: EA [-] 27.36±10.74 years; EA [+] 25,55±7.47 years [t (2, 262) =1.661, p< 0.09]. EA [+] were more likely to be male (40.4%) than EA [-] (27.3%) (Χ2=5.060, df=1, p=0.026). There were no differences in the levels of education (primary/secondary/ college student/ university student/ completed higher education) in the studied groups (Χ2=1.78, df=4, p=0.786). EA [+] showed a higher level of morningness measured with the CSM total score (34.13±6.48) than EA [-] (30.62±8.63)[t (2, 251.8) = 3.855, p< 0.001).

**Conclusions:**

EA was linked to male sex in the studied sample. Individuals with positive exercise addiction screen present higher levels of morningness.

**Disclosure of Interest:**

None Declared

